# Exploring the clinical utility of DPP-IV and SGLT2 inhibitors in papillary thyroid cancer: a literature review

**DOI:** 10.3389/fphar.2024.1323083

**Published:** 2024-01-16

**Authors:** Angelika Buczyńska, Maria Kościuszko, Adam Jacek Krętowski, Anna Popławska-Kita

**Affiliations:** ^1^ Clinical Research Centre, Medical University of Bialystok, Bialystok, Poland; ^2^ Department of Endocrinology, Diabetology and Internal Medicine, Medical University of Bialystok, Bialystok, Poland

**Keywords:** papillary thyroid cancer, DPP-IV, Isglt2, adjuvant therapy, thyroid cancer

## Abstract

In the realm of clinical management, Papillary Thyroid Cancer (PTC) stands out as a prevalent thyroid malignancy, characterized by significant metabolic challenges, particularly in the context of carbohydrate metabolism. Recent studies have unveiled promising applications of Dipeptidyl Peptidase-IV (DPP-IV) and Sodium-Glucose Cotransporter 2 (SGLT2) inhibitors, which are conventionally employed in the treatment of type 2 diabetes mellitus (T2DM), as potential adjuncts in anticancer therapy. DPP-IV and SGLT2 inhibitors can be imply to counteract the Warburg effect in cancer, with a specific focus on PTC, owing to their potential metabolic advantages and their influence on the tumor microenvironment, achieved by imposing restrictions on glucose accessibility. Consequently, a comprehensive review has been undertaken, involving meticulous examination of the existing body of evidence pertaining to the utilization of DPP-IV and SGLT2 inhibitors in the context of PTC. The mechanisms of action inherent to these inhibitors have been thoroughly explored, drawing upon insights derived from preclinical investigations. Furthermore, this review initiates discussions concerning the implications for future research directions and the formulation of innovative therapeutic strategies for PTC. As the intricate interplay between carbohydrate metabolism, the Warburg effect, and cancer progression garners increasing attention, attaining a comprehensive understanding of the roles played by DPP-IV and SGLT2 inhibitors in PTC management may serve as the cornerstone for novel approaches aimed at enhancing patient care and broadening the spectrum of available therapeutic modalities.

## 1 Introduction

Papillary Thyroid Cancer (PTC) is the most prevalent subtype of thyroid malignancy, accounting for approximately 80% of all thyroid cancer cases (Rego-Iraeta et al., 2009; Li et al., 2022). According to data from the Surveillance, Epidemiology, and End Results (SEER) database spanning from 1975 to 2012, the rate of PTC diagnoses rose from 4.8 to 14.9 per 100,000 individuals (K et al., 2020; Davies and Welch, 2006). It typically exhibits relatively favorable prognosis; however, the clinical management of PTC remains a complex and evolving challenge (Gorgone et al., 2009). Nevertheless, the clinical management of thyroid cancer depends on the specific risk of cancer progression and encompasses various approaches, such as total thyroidectomy, lobectomy, radioiodine therapy (RAI), laser ablation, or active surveillance based on 2015 American Thyroid Association guidance (Haugen et al., 2016). Currently, there is a lack of non-invasive pharmacological interventions that could potentially confer additional anti-cancer effects. This limitation restricts the utilization of non-invasive treatment options, necessitating reliance on invasive methods (Haugen et al., 2016; Cho et al., 2019). The latest literature data highlights that the regulation of carbohydrate metabolism constitutes an important and modern focal point in many anticancer therapies. Accordingly, the Warburg effect represents a significant area of research as the basis for anticancer action in various types of cancers, including thyroid cancer (Tran et al., 2016). Thus, recent research has shed light on the potential role of novel pharmacological medicaments, such as antihyperglycemic agents, in the treatment of PTC (Li et al., 2020). Among these agents, Dipeptidyl Peptidase-IV (DPP-IV) inhibitors and Sodium-Glucose Cotransporter 2 (SGLT2) inhibitors have emerged as promising candidates (Busek et al., 2022; Basak et al., 2023). DPP-IV and SGLT2 inhibitors are primarily known for their applications in the management of type 2 diabetes mellitus (T2DM). DPP-IV inhibitors prevent the degradation of incretin hormones, such as glucagon-like peptide-1 (GLP-1) and glucose-dependent insulinotropic polypeptide (GIP), resulting in improved glycemic control (Golightly et al., 2012). On the other hand, SGLT2 inhibitors reduce glucose reabsorption in the renal tubules, leading to glycosuria and lower blood glucose levels (Scheen, 2015). The link between T2DM and cancer has long been a subject of investigation, and recent studies have demonstrated that individuals with T2DM implicated in antihyperglycemic agents are characterized by decreased risk of certain malignancies, including PTC (Bea et al., 2023). Given the established roles of DPP-IV and SGLT2 inhibitors in diabetes management and their potential impact on cancer pathways, it is essential to explore their potential clinical utility in PTC.

This literature review aims to comprehensively explore the existing evidence regarding the use of DPP-IV and SGLT2 inhibitors in the context of PTC. By examining the mechanisms of action, preclinical studies, and clinical trials, we aim to elucidate the potential benefits and limitations of these pharmacological agents in the management of PTC. Additionally, we will address the implications of these findings for future research directions and potential therapeutic strategies for PTC patients. As the field of thyroid cancer research continues to advance, understanding the role of DPP-IV and SGLT2 inhibitors in potential PTC management may offer new avenues for improving patient outcomes and expanding the therapeutic armamentarium for this prevalent malignancy.

## 2 Materials and methods

To conduct this comprehensive literature review, a systematic approach encompassing several key steps was employed. Initially, a meticulous selection of pertinent scientific literature related to PTC and DPP-IV SGLT2 inhibitors was undertaken. This encompassed articles, reviews, and clinical studies available in databases such as PubMed, MEDLINE, and Google Scholar. Subsequently, inclusion and exclusion criteria were defined. Only peer-reviewed articles published in the English language were included, and studies encompassing both human and animal models, as well as preclinical and clinical investigations examining the impact of DPP-IV and SGLT2 inhibitors on cancer, especially PTC development, were considered. Conversely, studies not directly relevant to the topic, publications in languages other than English, and those inaccessible via reputable scientific databases were excluded from the review. The search strategy was constructed using a combination of keywords and Medical Subject Headings (MeSH), including terms such as “Papillary Thyroid Cancer,” “Thyroid Neoplasms,” “DPP-IV inhibitors,” “SGLT2 inhibitors,” and their variations. Boolean operators (AND, OR) were deployed to formulate precise search queries. Additionally, manual scrutiny of reference lists within selected articles was conducted to identify supplementary sources.

Data extraction involved the retrieval and structured organization of information from the chosen articles. This encompassed details pertaining to study design, patient characteristics, interventions (DPP-IV and SGLT2 inhibitors), outcomes, and conclusions. Particular emphasis was placed on comprehending the mechanisms of action of these inhibitors and their potential influence on PTC development. Qualitative synthesis of the literature was subsequently conducted, focusing on key findings, trends, and novel insights concerning the clinical potential of DPP-IV and SGLT2 inhibitors in the management of PTC. A critical appraisal of the included studies was also performed to assess methodological quality and identify potential sources of bias. Results and interpretations from the selected sources were synthesized and discussed within the context of the research objectives. Implications for future research directions and innovative therapeutic strategies were considered, underscoring the importance of exploring unconventional therapies to optimize outcomes in PTC treatment. The review concluded with a summary of the current state of knowledge in this field.

## 3 Papillary thyroid cancer risk factors

The incidence of PTC has been steadily increasing over the past few decades worldwide (Sung et al., 2021; Pizzato et al., 2022). PTC displays a significant gender bias, with females being roughly three times as susceptible to its development as males (Arroyo et al., 2022). The consistent gender disparity in PTC incidence has been a recurring observation in various populations and geographical contexts. Numerous investigations have explored plausible factors, including hormonal influences (notably, the presence of estrogen receptors in thyroid tissue, and estrogen’s potential role in stimulating thyroid cell growth and proliferation) and genetic factors, such as the BRAF mutation, as well as other genetic alterations like RET/PTC rearrangements and RAS mutations, which may underlie this gender difference (Leclair et al., 2021). Furthermore, PTC can impact individuals across all age brackets, although it is most frequently identified in adults ranging from 30 to 50 years old (Arroyo et al., 2022). Nonetheless, the occurrence of PTC demonstrates substantial variation among various nations and geographic areas. Elevated incidence rates have been documented in regions characterized by increased iodine consumption, particularly in areas with a high prevalence of goiter (Zhang et al., 2022). Current research endeavors to elucidate the influence of environmental factors, genetic predisposition, and disparities in healthcare practices as contributing factors to these geographical discrepancies (Miranda-Filho et al., 2021). Notably, there is a well-established association between exposure to ionizing radiation, particularly during childhood, and an increased risk of developing PTC (Su et al., 2016). Individuals who have received radiation therapy as a treatment for medical conditions, such as Hodgkin’s lymphoma or head and neck cancers, face an elevated likelihood of PTC occurrence (Albi et al., 2017; Buczyńska et al., 2023a). Numerous studies have consistently demonstrated a dose-response relationship between radiation exposure and the risk of PTC. A study conducted by Baloch et al. identified several noteworthy prognostic factors associated with PTC prognosis. These factors encompassed age, tumor size, the presence of lymph node metastasis, extrathyroidal extension, and distant metastasis. The findings indicated that older age, larger tumor size, the presence of lymph node metastasis, and extrathyroidal extension were correlated with a less favorable prognosis, whereas the presence of distant metastasis signified a notably worse outcome. This study provided valuable insights into prognostic factors, including the measurement of angioinvasion, which can aid in predicting the outcomes of patients diagnosed with well-differentiated follicular-derived carcinoma, specifically PTC (Baloch and LiVolsi, 2001; Buczyńska et al., 2023b; Buczyńska et al., 2023c). In light of the above findings, there is a need to continue exploring new, minimally invasive therapeutic approaches that will be well-tolerated within the PTC patient population.

## 4 Warburg effect–the novel target in PTC clinical management

**FIGURE 1:**
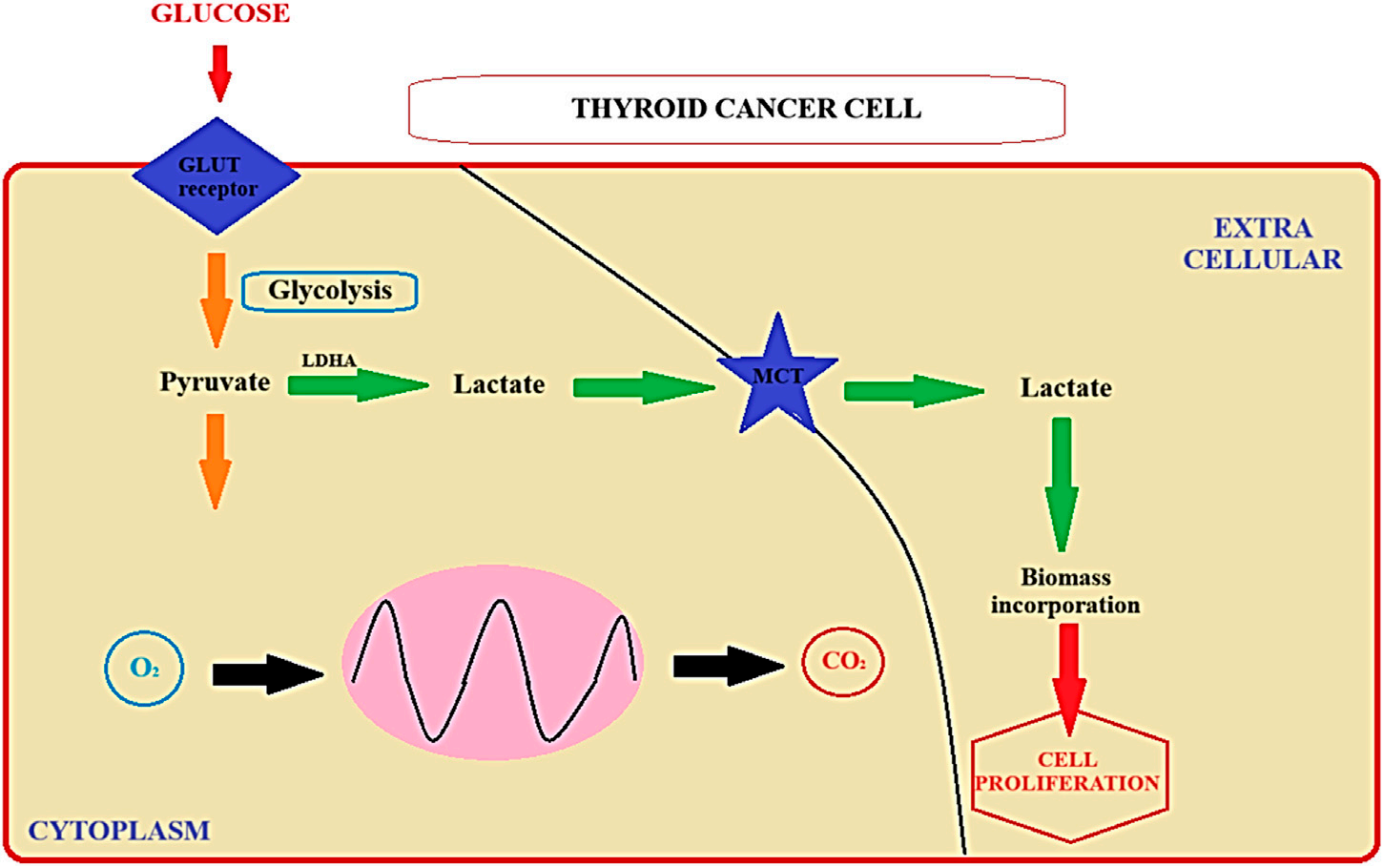
The Warburg effect (Liberti et al., 2016). LDHA - Lactate Dehydrogenase A; MCT-Medium-Chain Triglycerides; GLUT Receptor - Glucose Transporter Receptor.

Literature data has indicated that molecular alterations within signaling pathways, such as RAS, RAF, and mitogen-activated protein kinase (MAPK), play a role in the development of thyroid neoplasms (Soares et al., 2003). Additionally, previous investigations have revealed that thyroid cancer is characterized by enhanced glucose uptake, as detected by 18F-fluorodeoxyglucose positron emission tomography (18F-FDG PET), along with reduced radioiodine uptake capability (Lang and Law, 2011; Larg et al., 2019). In general, transformed cells exhibit modified energy metabolism, as they do not inhibit glycolysis in the presence of oxygen. Nearly a century ago, Warburg observed that cancer cells can convert glucose into lactate even when oxygen is available, a phenomenon termed aerobic glycolysis or the “Warburg effect” (Liberti et al., 2016). Subsequent research has demonstrated increased expression of glucose transporters (GLUTs) alongside decreased oxidative metabolism in cancer cells (Tran et al., 2016; Kościuszko et al., 2023). While the Warburg hypothesis is well-established, the precise adaptive mechanisms responsible for reduced oxidative metabolism in cancer cells remain unclear in many carcinoma models (Schiliro et al., 2021). These characteristics underscore the necessity of investigating the metabolic profile of thyroid tumors (Lee et al., 2015). Despite their representing an intriguing spectrum of tumors with varying prognoses, few studies have directly assessed energy metabolism in relation to tumorigenesis (Martínez-Reyes and Chandel, 2021). Skorupa et al. recently demonstrated that thyroid iodide and glucose uptake are influenced by the AMP-activated protein kinase (AMPK) signaling pathway, often referred to as the cellular energy “fuel gauge” (Skorupa et al., 2021). However, the unique metabolic characteristics of thyroid cancer cells do not merely represent a passive alteration but rather constitute an active change in genetic expression, leading to a shift in metabolic pathways that promote the development and invasiveness of tumors (Heydarzadeh et al., 2020). The study performed by Matsuzu et al. showed that in all thyroid parenchymal cells, there was uniform expression of GLUT1, GLUT3, GLUT4, and GLUT10. GLUT1 exhibited heightened expression in carcinoma cases, as well as when compared to corresponding normal tissue samples from the same patient. The expression levels of other GLUTs remained statistically unchanged in pathological tissues. These findings support the hypothesis that during the development of cancer in the thyroid, GLUT1 becomes overexpressed and could have a significant impact on increased glucose absorption by thyroid cancer cells (Matsuzu et al., 2004). Therefore, high expression of GLUT1, positively correlate with the proliferative index and equates to the malignant characteristic (Morani et al., 2014). Thus, targeting tumor metabolism holds promise as a potential approach for treating thyroid cancer. Additionally, molecules associated with tumor metabolism may serve as prognostic markers for thyroid cancer outcomes (Rogucki et al., 2021; Rogucki et al., 2022). Prior research has linked thyroid cancer progression to increased glucose uptake. These findings suggest that pivotal molecules involved in tumor Warburg effect and metabolism could play a role in predicting the prognosis of thyroid cancer simultaneously defining novel medical targets. The pathomechanism of the Warburg effect, along with the identification of potential medical targets, has been delineated in Figure 1 (Figure 1).

## 5 Antihyperglycemic agents in the treatment of thyroid cancer

Metformin is a commonly prescribed medication for diabetes management, and it is known for its excellent safety record. Over the past decade, several studies have indicated that metformin can lower thyroid-stimulating hormone (TSH) levels in individuals with diabetes. Most research suggests that metformin can reduce TSH levels in individuals who have either overt or subclinical thyroid issues, but this effect is not observed in people with normal thyroid function (Pappa and Alevizaki, 2013; Adamska et al., 2021; Buczyńska et al., 2021; Buczyńska et al., 2022). Additionally, metformin seems to possess anti-growth properties against various types of thyroid cancer (Wang et al., 2022a). However, there is experimental evidence suggesting that metformin may reduce the effectiveness of radioactive iodine treatment in individuals with differentiated thyroid cancer. This limitation might restrict its use in the management of such cancer cases (Pappa and Alevizaki, 2013). Consequently, exploring alternative antihyperglycemic drugs like DPP-IV and SGLT2 inhibitors is of significant interest for PTC patients. To succinctly encapsulate the key findings of the most significant studies, Table 1 has been generated (Table 1).

**TABLE 1:** The sSummary of kKey fFindings from rRelevant PTC sStudies.

Study type of intervention examination	Participants	Method of detection	Outcomes	Reference
DPP-IV	18 thyroid carcinoma tissues and paracancerous tissues and 12 medullary thyroid carcinoma tissues	immunohistochemistry	DPP-IV mRNA expression was significantly higher in MTC thyroid carcinoma tissues vs. thyroid carcinoma tisues	Gao et al. (2022)
			Higher DPP-IV expression was linked with poorer prognosis	
	N/D	bioinformatics analysis	overexpression of the DPP4/CTNNB1/MET gene is associated with immuno-invasive phenotypes, cancer progression, metastasis, resistance to treatment, and unfavorable clinical outcomes	Cheng et al. (2022)
			DPP-IV inhibitors were designated for these patients	
	mouse xenograft model	immunohistochemistry	high DPP-IV expression was significantly linked to extrathyroidal extension, BRAF mutations, and advanced tumor stages	Lee et al. (2017)
			silencing DPP-IV or using DPP-IV inhibitors significantly reduced colony formation, cell migration, and invasion	
			sitagliptin treatment reduced tumor growth and the expression of transforming growth factor-β receptor I in xenografts mouses	
	GLAG-66 and TPC-1 PTC tissues	silencing the DPP-IV gene using siRNA/DPP-IV inhibitor	Using siRNA or the DPP-IV inhibitor sitagliptin led to a substantial reduction in the expression of key factors associated with PTC pathogenesis; reduced expression and phosphorylation of ERK1/2, JNK1, and P38 MAPK were observed, indicating modulation of the MAPK pathway; Decreased expression of pro-angiogenic factors such as VEGF, along with downregulation of fibroblast growth factor receptors (FGFR-1), TGF-β1, Snail, and HIF-1α, suggests an anti-angiogenic effect; an increase in the expression of cell adhesion protein E-cadherin and apoptosis-related protein Bax was noted, indicating a potential influence on cellular processes	Hu et al. (2021)
SGLT2	thyroid cancer cells both *in vitro* and *in vivo*	immunohistochemistry and clinical dataset analysis	Inhibition of SGLT2 mitigates thyroid cancer cell proliferation *in vitro* and *in vivo*	Wang et al. (2022b)
			Canagliflozin, an SGLT2 inhibitor, suppresses glucose uptake and glycolysis in thyroid cancer cells	
			Canagliflozin inhibits the AKT/mTOR signaling pathway and enhances AMPK activation	
			Canagliflozin hinders the G1 to S phase transition in the cell cycle, downregulating cyclin D1, cyclin D3, cyclin E1, cyclin E2, and E2F1	
			Canagliflozin induces apoptosis in thyroid cancer cells	
			Canagliflozin activates the DNA damage response pathway ATM/CHK2 and increases γ-H2AX expression	
			Elevated SGLT2 levels observed in thyroid cancer tissues of patients	
			Positive correlation between SGLT2 expression and cyclin D3 levels in thyroid cancer patients	
			Inhibition of SGLT2 may restrict PTC development by reducing cyclin D1 and D3 expression, inhibiting excessive cell proliferation	

AKT/mTOR, serine/threonine protein kinase/Mammalian Target of Rapamycin; AMPK - AMP-Activated Protein Kinase; ATM/CHK2 - Ataxia Telangiectasia Mutated/Checkpoint Kinase 2; Bax - Bcl-2-associated X protein; Canagliflozin - SGLT2, inhibitor; DPP-IV, Dipeptidyl Peptidase IV; E-cadherin - Epithelial Cadherin; ERK1/2 - Extracellular Signal-Regulated Kinase 1/2; FGFR-1, Fibroblast Growth Factor Receptor 1; G1 to S phase - Transition from the Gap 1 phase to the Synthesis phase; GLAG-66, type of PTC, tissue; HIF-1α - Hypoxia-Inducible Factor 1 Alpha; JNK1 - c-Jun N-terminal Kinase 1; MAPK, Mitogen-Activated Protein Kinase; MTC, Medullary Thyroid Carcinoma; P38 MAPK, p38 Mitogen-Activated Protein Kinase; N/D–No data; PTC, Papillary Thyroid Carcinoma; SGLT2 - Sodium-Glucose Co-Transporter 2; siRNA, Small Interfering RNA; TPC-1 PTC, Thyroid Papillary Carcinoma; TPC-1, cell line; TGF-β1, Transforming Growth Factor Beta 1; VEGF, Vascular Endothelial Growth Factor; γ-H2AX, Phosphorylated form of Histone H2AX.

### 5.1 DPP-IV inhibitors

The scientific basis for their therapeutic utility in the context of cancer treatment lies in their multifaceted actions on various physiological processes. DPP-IV is an enzyme responsible for the degradation of incretin hormones such as glucagon-like peptide-1 (GLP-1) and glucose-dependent insulinotropic peptide (GIP). These hormones play pivotal roles in regulating glucose homeostasis and insulin secretion. DPP-IV inhibitors, by virtue of their mechanism of action, increase the bioavailability of GLP-1 and GIP, leading to enhanced insulin secretion, improved glycemic control, and reduced insulin resistance in diabetes. Beyond their antidiabetic effects, DPP-IV inhibitors have exhibited additional properties that make them potentially valuable in oncology. During DPP-IV inhibition by substance P inhibition of synthesis the expression of GLUT4 in thyroid cancer cells would be reduced (Barchetta et al., 2022). Moreover, DPP-IV is expressed on various immune cells, including T lymphocytes. Inhibition of DPP-IV has been associated with immunomodulatory effects, resulting in enhanced T-cell function and immune responses. This aspect is of particular interest in cancer therapy, where a robust immune response against tumor cells is crucial for effective eradication (Shao et al., 2020). Inflammation is a recognized contributor to cancer progression and metastasis, making the anti-inflammatory potential of DPP-IV inhibitors a valuable asset in cancer treatment. Additionally, DPP-IV has been implicated in angiogenesis, a process pivotal for tumor growth and metastasis. Inhibition of DPP-IV may impede angiogenesis by altering the availability of angiogenic factors, further contributing to its potential antitumor effects. In preclinical studies and some clinical trials, DPP-IV inhibitors have exhibited promise in reducing tumor growth and enhancing the efficacy of conventional cancer therapies (Enz et al., 2019).

#### 5.1.1 Potential implication in PTC clinical management

**FIGURE 2:**
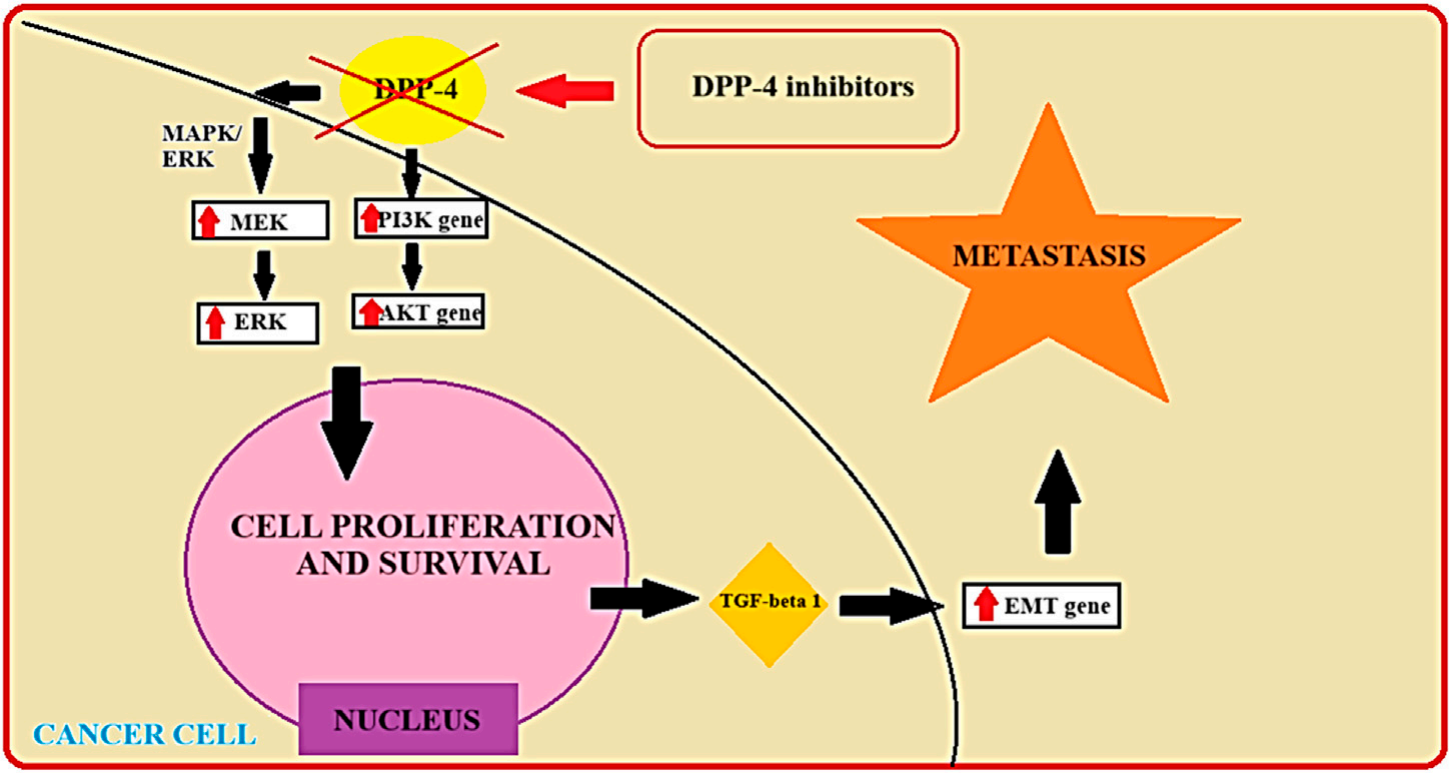
The potential targets of DPP-IV among PTC cells (Deacon, 2018). AKT - serine/threonine protein kinase; DPP-4 - Dipeptidyl Peptidase-4; EMT - Epithelial-Mesenchymal Transition Gene; ERK - Extracellular Signal-Regulated Kinase; MAPK/ERK - Mitogen-Activated Protein Kinase/Extracellular Signal-Regulated Kinase; MEK - Mitogen-Activated Protein Kinase; PI3K Gene - Phosphoinositide 3-Kinase Gene; TGF-beta 1 - Transforming Growth Factor-beta 1.

Preclinical studies and emerging clinical evidence suggest that DPP-IV inhibitors may exert direct anti-tumor effects in PTC (Lee et al., 2017). The schematic depiction of the DPP-IV inhibitor’s mechanism, coupled with the identification of potential targets within differentiated thyroid cancer, has been incorporated in Figure 2 (Figure 2).

Gao et al. conducted a study on DPP-IV in thyroid carcinoma (THCA) and its links to prognosis and antitumor immunity. They analyzed data from The Cancer Genome Atlas (TCGA), Genotype-Tissue Expression (GTEx), and Gene Expression Omnibus (GEO) databases to explore DPP-IV expression, its impact on THCA prognosis, and its connection to immune response. Additionally, they validated DPP-IV mRNA levels in 18 THCA tissues and paracancerous tissues and confirmed DPP-IV protein levels in 12 medullary thyroid carcinoma (MTC) tissues using immunohistochemistry. Their bioinformatics analysis revealed that DPP-IV mRNA expression was significantly higher in THCA than in paracancerous tissues, with the highest expression in MTC. They observed differences in DPP-IV expression based on clinical characteristics. Higher DPP-IV expression in THCA was linked to lower disease-free survival (HR = 1.8). The study also showed a substantial connection between DPP-IV, immune cell infiltration, immune response, and 21 immune checkpoint genes (ICGs) in THCA. Laboratory results demonstrated significant upregulation of DPP-IV mRNA in 18 THCA tissues compared to paracancerous tissues. Immunohistochemistry indicated higher DPP-IV protein levels in 12 MTC tissues compared to paracancerous tissues. These effects might involve tumor cell proliferation suppression, apoptosis induction, or interference with PTC progression signaling pathways. DPP-IV inhibitors have shown promise in modulating the immune response, particularly by enhancing T-cell function. This immunomodulatory property is crucial in cancer therapy, potentially enhancing treatment efficacy when combined with conventional treatments like surgery, radioactive iodine therapy, or targeted therapies. Research on the safety and efficacy of such combinations is a critical area of study (Gao et al., 2022). In the similar study performed by Cheng et al., a bioinformatics analysis was employed to identify crucial theragnostic markers for thyroid carcinoma (THCA). Remarkably, the analysis revealed the overexpression of the DPP4/CTNNB1/MET gene signature in PTCa. These findings suggest that this signature is associated with immuno-invasive phenotypes, cancer progression, metastasis, resistance to treatment, and unfavorable clinical outcomes in thyroid cancer patients. Given the limitations of conventional cancer drugs, which can be cytotoxic and non-specific, decided to investigate the potential anticancer effects of the antidiabetic drug sitagliptin, which has recently shown promise as an anticancer agent and is known for its good tolerability and efficacy. Interestingly, their *in silico* molecular docking results indicated that sitagliptin may exhibit putative binding affinities with the DPP4/CTNNB1/MET signatures, surpassing those of standard inhibitors for these genes. This suggests that sitagliptin holds promise as a potential therapeutic option for THCA, warranting further investigation in both *in vitro* and *in vivo* studies, as well as in clinical settings (Cheng et al., 2022).

Therefore, Jie-Jen et al. conducted a study to investigate the role of DPP-IV in thyroid cancer and its underlying mechanisms. Previous research had suggested that DPP-IV could be a marker for thyroid cancer, but its prognostic significance was unclear. They used immunohistochemistry to assess DPP-IV expression in thyroid cancer tissue microarrays. In addition, they conducted *in vitro* studies with genetic and pharmacological DPP-IV inhibition. Gene expression and pathway analyses were used to identify molecular targets affected by DPP-IV. They also evaluated the therapeutic potential of DPP-IV inhibition using a mouse xenograft model. The study found that high DPP-IV expression was significantly linked to extrathyroidal extension, BRAF mutations, and advanced tumor stages in PTC. Individuals with high DPP-IV expression were less likely to achieve a “no evidence of disease” status during follow-up. *In vitro* experiments showed that silencing DPP-IV or using DPP-IV inhibitors significantly reduced colony formation, cell migration, and invasion. Analysis of gene expression changes after DPP-IV knockdown suggested involvement of the transforming growth factor-β signaling pathway. *In vivo* experiments demonstrated that sitagliptin treatment reduced tumor growth and the expression of transforming growth factor-β receptor I in xenografts (Lee et al., 2017).

In the following study performed by Hu et al. aimed to comprehensively examine the role of the DPP-IV gene in PTC tissues and its potential association with the MAPK pathway the significant increase in DPP-IV gene expression in PTC tissues were observed. Experiments conducted on PTC cell lines (GLAG-66 and TPC-1) demonstrated that silencing the DPP-IV gene using siRNA or employing the DPP-IV inhibitor, sitagliptin, resulted in a substantial reduction in the expression of key factors related to PTC pathogenesis. Specifically, reduced expression and phosphorylation of ERK1/2, JNK1, and P38 MAPK were observed, along with decreased expression of pro-angiogenic factors (VEGF), fibroblast growth factor receptors (FGFR-1), transforming growth factor β1 (TGF-β1), Snail, and hypoxia-inducible factor 1α (HIF-1α). Simultaneously, an increase in the expression of cell adhesion (E-cadherin) and apoptosis-related (Bax) proteins was noted. Furthermore, DPP-IV gene silencing inhibited PTC cell proliferation and enhanced apoptosis. The findings from this study suggest that the silencing of the DPP-IV gene exerts a significant impact on the molecular and cellular processes associated with PTC progression, primarily through modulation of the MAPK pathway and the expression of key regulatory factors. These discoveries may open new avenues for understanding and treating PTC (Hu et al., 2021).

#### 5.1.2 Future perspectives

Identifying predictive biomarkers that can stratify PTC patients who are likely to benefit from DPP-IV inhibitor therapy is essential. These biomarkers may encompass molecular signatures, genetic profiles, or immunological markers that can guide patient selection and personalized treatment strategies. Conducting well-designed clinical trials specifically focused on the use of DPP-IV inhibitors in PTC is imperative. These trials should assess the safety, efficacy, and long-term outcomes of DPP-IV inhibitor-based regimens in PTC patients. Robust clinical data are essential to establish their role in routine clinical practice.

### 5.2 SGLT2 inhibitors

The exploration of SGLT2 inhibitors in the context of antitumor treatment represents an intriguing avenue of investigation with several scientific implications. Originally developed for their glucose-lowering properties in diabetes management, SGLT2 inhibitors have displayed multifaceted effects that may have relevance in the broader field of cancer therapy (Dutka et al., 2022).

**FIGURE 3:**
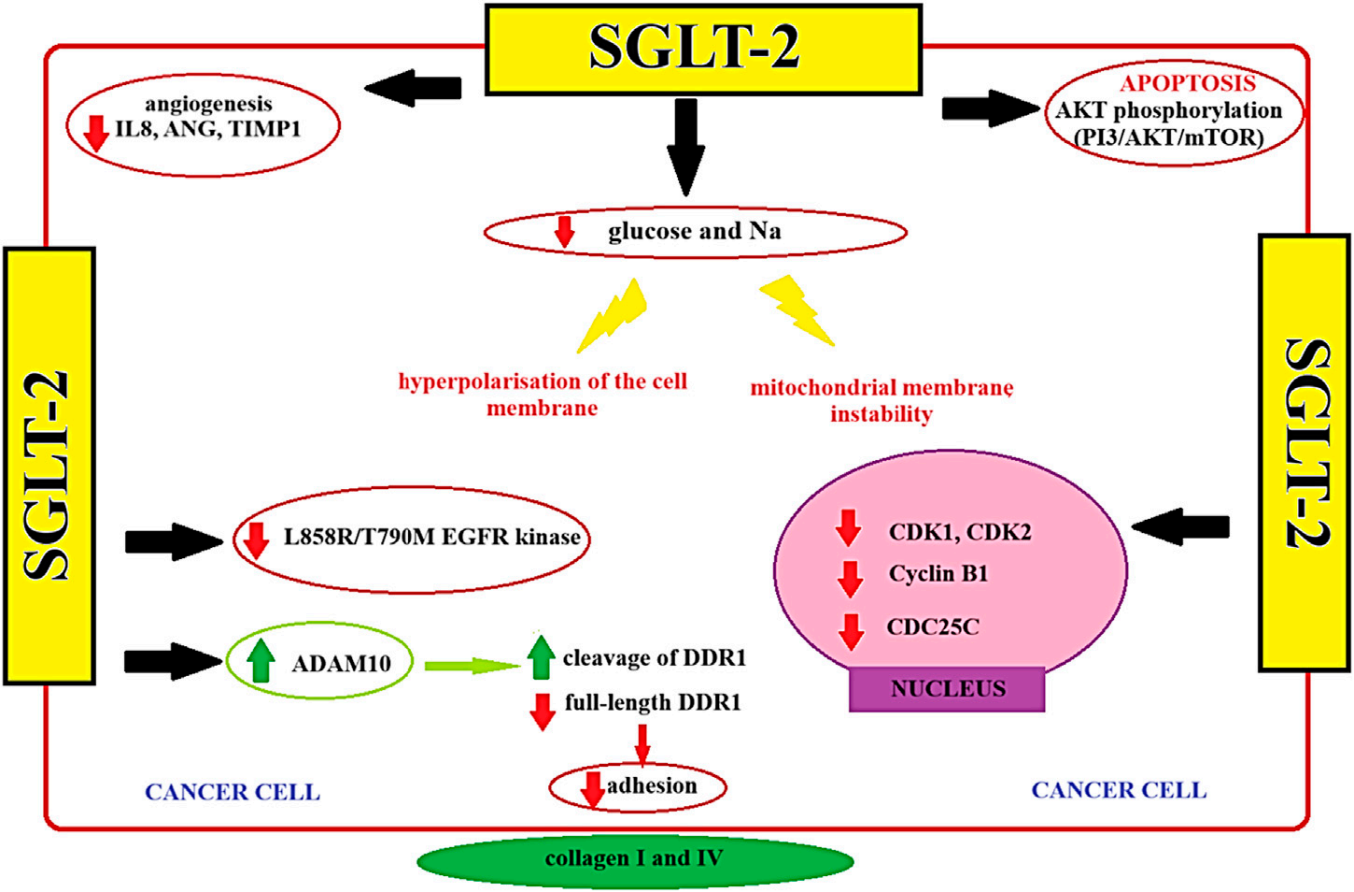
The potential targets of SGLT-2 among PTC cells (Wang et al., 2022b). AKT - serine/threonine protein kinase; ADAM10 - A Disintegrin and Metalloproteinase 10; ANG–Angiopoietin; CDC25C - Cell Division Cycle 25C; CDK1 - Cyclin-Dependent Kinase 1 CDK2 - Cyclin-Dependent Kinase 2; Cyclin B1 - Cyclin B1; DDR1 - Discoidin Domain Receptor 1 IL8 - Interleukin 8; L858R/T790M EGFR Kinase - Leucine 858 to Arginine/Threonine 790 to Methionine Epidermal Growth Factor Receptor Kinase; mTOR - Mechanistic Target of Rapamycin; PI3 - Phosphoinositide 3-Kinase; SGLT2 - Sodium-Glucose Transporter 2; TIMP1 - Tissue Inhibitor of Metalloproteinases 1.

SGLT2 inhibitors have been associated with significant metabolic alterations, including reductions in blood glucose levels, insulin resistance, and body weight. These metabolic changes could potentially impact the tumor microenvironment, affecting factors such as nutrient availability and oxidative stress, which are crucial for tumor cell survival and proliferation (Wright, 2021). Emerging evidence suggests that SGLT2 inhibitors may influence various cellular signaling pathways that are implicated in cancer progression (Benedetti et al., 2022; L et al., 2014). This modulation could potentially lead to the inhibition of tumor growth or the sensitization of tumor cells to other antitumor agents. The tumor microenvironment plays a pivotal role in cancer development and progression. SGLT2 inhibitors may affect the tumor microenvironment by altering the composition of immune cells, cytokine profiles, and angiogenesis. Investigating these potential effects in the context of antitumor treatment is crucial (Basak et al., 2023; L et al., 2014; Bose et al., 2021). Given their metabolic and signaling effects, SGLT2 inhibitors may have synergy with existing cancer therapies, such as chemotherapy, immunotherapy, or targeted therapies. In the study performed by Xie et al. identified that SGLT2 inhibitor possesses the ability to inhibit the advancement and progression of cervical cancer. This inhibitory effect is attributed to the activation of the adenosine monophosphate (AMP)-activated protein kinase (AMPK) signaling pathway and the concurrent downregulation of forkhead Box A1 (FOXA1) and sonic hedgehog (SHH) expression (Xie et al., 2020). *In vitro*, SGLT2 exhibited antitumor activity by augmenting caspase 3 cleavage activity and modulating the expression of Bax/Bcl-2 (Xie et al., 2020). The following study performed by Faridi et al. investigated SGLT2 inhibitors impact on the human lung cancer cell line A549, revealing a significant inhibitory effect. A computer simulation study using molecular docking software identified apoptosis-related proteins, including Bcl-2, p53, and Caspase-3, as potential targets for SGLT2 inhibitor. The study indicated favorable binding with apoptotic protein receptors 1GJH, 1TUP, and 2XYG, suggesting a potential binding affinity between SGLT2 inhibitors and these receptors (Faridi et al., 2020). Furthermore, SGLT2 inhibitors may be used as adjuvant cancer treatment leading to better treatment outcomes. The study performed by focuses on overcoming challenges in treating triple-negative breast cancer (TNBC), a highly aggressive and drug-resistant subtype. Researchers explore the potential of the PI3K/AKT/mTOR pathway as a target for TNBC, finding improved prognosis with AKT inhibitors combined with paclitaxel. Doxorubicin (DOX), a common TNBC chemotherapy, faces resistance issues. SGLT2 inhibitors emerges as a promising candidate to sensitize tumor cells to DOX, offering cardioprotective effects and promoting apoptosis. Combining SGLT2 inhibitor with DOX synergistically inhibits TNBC cell survival by interfering with the mTOR pathway and inhibiting calmodulin. SGLT2 inhibitor significance lies in its ability to sensitize TNBC cells to DOX, offering a potential strategy to enhance chemotherapy efficacy and overcome drug resistance in this challenging cancer subtype. Combining SGLT2 inhibitors with conventional treatments could enhance treatment efficacy and improve patient outcomes (Basak et al., 2023). The diagram illustrating the operational dynamics of the SGLT2 inhibitor, alongside the identification of prospective targets in differentiated thyroid cancer, has been presented in Figure 3 (Figure 3).

#### 5.2.1 Potential implication in PTC clinical management

The study performed by Wang et al. employed a multifaceted approach to investigate the impact of SGLT2 levels and the potential anticancer effects of canagliflozin in the context of PTC. Inhibition of SGLT2 demonstrated a mitigating effect on the proliferation of thyroid cancer cells both *in vitro* and *in vivo*. Canagliflozin, an SGLT2 inhibitor, exhibited several notable effects on thyroid cancer cells. It suppressed glucose uptake, glycolysis, and the activation of the AKT/mTOR signaling pathway while concurrently enhancing AMPK activation within the thyroid cancer cells. Moreover, canagliflozin hindered the transition from the G1 to S phase in the cell cycle and downregulated the expression levels of key cell cycle regulators, including cyclin D1, cyclin D3, cyclin E1, cyclin E2, and E2F1. Additionally, canagliflozin induced apoptosis in thyroid cancer cells. Further exploration uncovered that canagliflozin had the capability to increase the expression of γ-H2AX, a marker of DNA damage, and activated the DNA damage response signaling pathway ATM/CHK2. In thyroid cancer patients, elevated levels of SGLT2 were observed in thyroid cancer tissues, and there was a positive correlation between SGLT2 expression and cyclin D3 levels (Wang et al., 2022b). Elevated levels of cyclin D3 may be indicative of increased cell proliferation in PTC (Romitti et al., 2016). Therefore, the inhibition of SGLT2 may potentially lead to the restriction of the development of PTC by reducing the expression levels of cyclin D1 and D3, thus inhibiting excessive cancer cell proliferation (Romitti et al., 2016; Wang et al., 2022b; Cai et al., 2023).

#### 5.2.2 Future perspectives

Conducting well-designed multiple clinical trials specifically focused on the use of SGLT2 inhibitors in cancer treatment is imperative. These trials should evaluate the safety, efficacy, and long-term outcomes of SGLT2 inhibitor-based regimens in PTC and different stages. SGLT2 inhibitors have shown cardiovascular benefits in patients with diabetes. Since cardiovascular disease often coexists with cancer, the cardiovascular effects of SGLT2 inhibitors may have additional relevance in cancer patients, especially those undergoing cancer treatments with potential cardiovascular risks. Identifying specific patient populations within the realm of oncology who may derive the most benefit from SGLT2 inhibitor therapy is a crucial consideration. This may involve identifying biomarkers, genetic profiles, or tumor characteristics that can guide treatment decisions.

## 6 Exploring limitations in the use of DPP-IV and SGLT2 inhibitors

These inhibitors, while holding promise in various therapeutic applications, are not without constraints. One notable limitation involves the need for a more comprehensive understanding of their long-term effects, particularly in populations with pre-existing conditions. Additionally, issues related to cost, patient adherence, and potential side effects contribute to the nuanced landscape of their practical implementation (Ahrén, 2010).

DPP-IV inhibitors demonstrate favorable safety and tolerability in extensive phase III clinical studies, with nasopharyngitis and cutaneous lesions as primary adverse events (Scheen, 2018; Sesti et al., 2019). Notably, these events rarely lead to treatment discontinuation. The efficacy and safety of DPP-IV inhibitors prove advantageous for individuals with renal impairment and elderly subjects with type 2 diabetes. Post-marketing surveillance and long-term cardiovascular safety studies reveal no significant safety imbalances (Gallwitz, 2016). Concerns about pancreatic safety prompted thorough evaluations by regulatory agencies, including the European Medicines Agency (EMA) and the US Food and Drug Administration (FDA) (Elashoff et al., 2011; Pinto et al., 2018). No causal link was found between incretin-based therapies, such as DPP-IV inhibitors, and pancreatic safety. Despite an acknowledged twofold risk of acute pancreatitis in type 2 diabetes, inclusion of an acute pancreatitis risk label, and low risk in retrospective studies, large cardiovascular safety studies did not confirm significant signals. An analysis estimated a number needed to harm of 1,066 for DPP-IV inhibitor therapy (Abbas et al., 2016). Recent reviews found no elevated cancer risk, including pancreatic cancer (Abbas et al., 2016). Concerning cutaneous manifestations, specifically bullous pemphigoid, retrospective analysis of over 9,000 patients treated in Japan (2009–2017) associated it with DPP-IV inhibitors, particularly vildagliptin (Kawaguchi et al., 2019). Regulatory agencies (EMA and FDA) imposed relevant labels (Kawaguchi et al., 2019). However, large cardiovascular safety studies did not confirm this association, warranting further investigation. Pathophysiologically, these skin lesions may be an “indirect target” effect of DPP-IV inhibitors.

Concerning SGLT2 inhibitors evaluation, the findings indicate that when compared to a placebo, the use of SGLT2 inhibitors did not demonstrate a significant difference in the risks associated with various adverse events. Specifically, there were similar incidences of hypoglycemia, urinary tract infections (UTI), genital infections, hypovolemia, and fractures observed between individuals treated with SGLT2 inhibitors and those administered a placebo (Mukai et al., 2021). The following study, incorporating data from six trials with 49,875 participants assessing four SGLT2 inhibitors, revealed a reduction in serious hyperkalemia risk (1754 cases) with a hazard ratio of 0.84 (95% CI, 0.76-0.93). This effect remained consistent across diverse studies (Pheterogeneity = 0.71). Investigator-reported hyperkalemia events (1,119 cases) also showed a lower incidence with SGLT2 inhibitors (hazard ratio, 0.80 [95% CI, 0.68-0.93]; Pheterogeneity = 0.21). This risk reduction extended across subgroups, including various baseline factors and medication use. Importantly, SGLT2 inhibitors did not increase the risk of hypokalemia (hazard ratio, 1.04 [95% CI, 0.94-1.15]; Pheterogeneity = 0.42). In conclusion, SGLT2 inhibitors mitigate serious hyperkalemia risk without elevating the risk of hypokalemia in individuals with type 2 diabetes, high cardiovascular risk, or chronic kidney disease (Neuen et al., 2022).

## 7 Conclusion

This literature review has illuminated the potential roles of DPP-IV and SGLT2 inhibitors in the management of PTC, albeit with limited exploration thus far. While promising mechanistic connections have been identified, their clinical utility remains uncertain in the absence of robust clinical trials. Of particular significance, both SGLT2 and DPP-IV inhibitors exhibit promise in cancer therapy, potentially disrupting critical cancer-related processes such as the Warburg effect. The modulation of glycolytic pathways and the tumor microenvironment underscores their anticancer potential. To advance our understanding of the therapeutic potential of these inhibitors, meticulous clinical trials are imperative. These trials should comprehensively assess safety, efficacy, and personalized applicability in PTC patients. Furthermore, such efforts may lead to innovative therapeutic strategies that could significantly enhance PTC management. This review highlights the promising yet untapped potential of DPP-IV and SGLT2 inhibitors in the context of PTC. While mechanistic links to cancer pathophysiology are evident, realizing their clinical benefits necessitates rigorous clinical validation. Moving forward, a collaborative, multicenter, research-driven approach will be crucial to unlock the therapeutic potential of these agents, potentially revolutionizing PTC treatment.

## Data Availability

The original contributions presented in the study are included in the article/Supplementary material, further inquiries can be directed to the corresponding authors.
